# Doege-potter syndrome: a report of a histologically benign but clinically malignant case

**DOI:** 10.1186/s13019-017-0630-4

**Published:** 2017-08-07

**Authors:** Do Wan Kim, Kook Joo Na, Ju Sik Yun, Sang Yun Song

**Affiliations:** Department of Thoracic and Cardiovascular Surgery, Chonnam National University Hwasun Hospital, Chonnam National University Medical School, 322 Seoyang-ro, Hwasun, 519-763 Korea

**Keywords:** Pleural disease, Tumor, Benign, Fibrous neoplasm

## Abstract

**Background:**

Solitary fibrous tumors of the pleura (SFTPs) are relatively rare tumors that originate from mesenchymal cells of submesothelial tissue of the pleura. Most patients with SFTPs are asymptomatic; however, pleuritic chest pain, cough, and dyspnea can develop. If hypoglycemia is associated with a solitary fibrous tumor, it is referred to as the Doege-Potter syndrome.

**Case presentation:**

A 70-year-old man had visited our hospital with a chief complaint of dyspnea, and he was diagnosed as having a solitary fibrous tumor. A few years later, he developed hypoglycemia, and he underwent excision of the mass.

**Conclusion:**

Occasionally, SFTPs induce several paraneoplastic events, such as hypertrophic osteoarthropathy. We described here a patient with an SFTP with Doege-Potter syndrome who was successfully treated with complete resection. Although lesions can be histologically benign, they can clinically present with malignant features.

## Background

Solitary fibrous tumors of the pleura (SFTPs) are rare neoplasms that originate from the visceral pleura. If hypoglycemia is associated with a solitary fibrous tumor, it is referred to as the Doege-Potter syndrome [1].

Occasionally, histologically benign tumors can clinically present as malignant tumors [2, 3]. We describe an operative case of a patient with Doege-Potter syndrome that significantly interfered with his activities of daily living, although it was not malignant.

## Case presentation

**Fig. 1:**
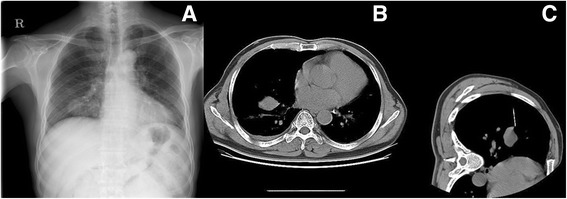
Chest radiograph on first diagnostic period. (**a**) Shows the mass shadow occupying middle and lower zone of right hemithorax. (**b**) 3.9 × 3.6 cm sized intrathoracic mass in right lower lobe lung field on Chest CT. (**c**) Biopsy CT during transthoracic needle biopsy

**Fig. 2:**
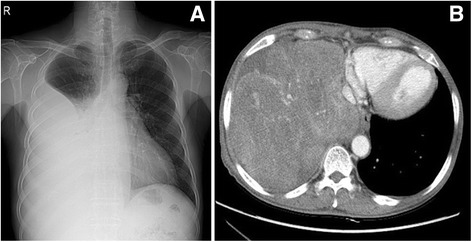
Chest radiograph on surgical treatment period. (**a**) Shows the increasing mass shadow occupying middle and lower zone of right hemithorax. (**b**) Chest CT during evaluation of lung mass shows lobulated heterogenously enhancing mass in right lower lobe lung field

A 70-year-old man had visited our hospital with a chief complaint of dyspnea in 2004, and at that time, a 3.9 × 3.6-cm tumor in the right hemithorax was detected The tumor was diagnosed as a pulmonary solitary fibrous tumor by transthoracic needle biopsy (Fig. 1 a-c). However, the patient was lost to follow-up. In April 2011, he visited our hospital again with complaints of acute shortness of breath. At that time, a large amount of pleural effusion and a 17 × 15-cm huge mass were observed on the chest radiograph, and a transthoracic needle biopsy and thoracentesis were performed accordingly. The tumor was diagnosed again as a solitary fibrous tumor, and surgical resection of the tumor was recommended. However, the patient refused to undergo the operation. When he visited our hospital to undergo positron emission tomography/computed tomography (CT), loss of consciousness developed suddenly while a blood sample was taken in the fasting state. Thus, he was admitted to the emergency room, and his blood glucose level was 38 mg/dL on admission. An examination of his medical history showed that the medication for diabetes mellitus was started 4 years ago, and this medication was stopped 2 years ago. According to the most recent chest CT scan, the size of the solitary fibrous tumor in the right hemithorax had increased to 18.5 × 14.5 cm, and a large amount of pleural effusion was observed (Fig. 2 a-b). After injecting 50 cm^3^ of 50% dextrose/water (D/W), his blood glucose level increased and consciousness recovered. To prevent the recurrence of hypoglycemia, 10% D/W was continuously injected, but hypoglycemia and loss of consciousness developed three more times over 3 days after admission to an inpatient ward. A hormone test was conducted to differentiate the cause of hypoglycemia. Although, no abnormal findings were observed and the tumor had enlarged and the blood glucose level decreased despite the patient’s history of diabetes, so the possibility of a hormonal etiology was excluded. Therefore, Doege-Potter syndrome was suspected, and accordingly, complete excision of the mass was performed through thoracotomy 8 days after admission under general anesthesia. Intraoperatively, the following findings were observed: adhesion of the tumor to the lung and inferior vena cava, a hypervascular lesion on the mass, and adhesion of the tumor to the lung and inferior vena cava.

**Fig. 3:**
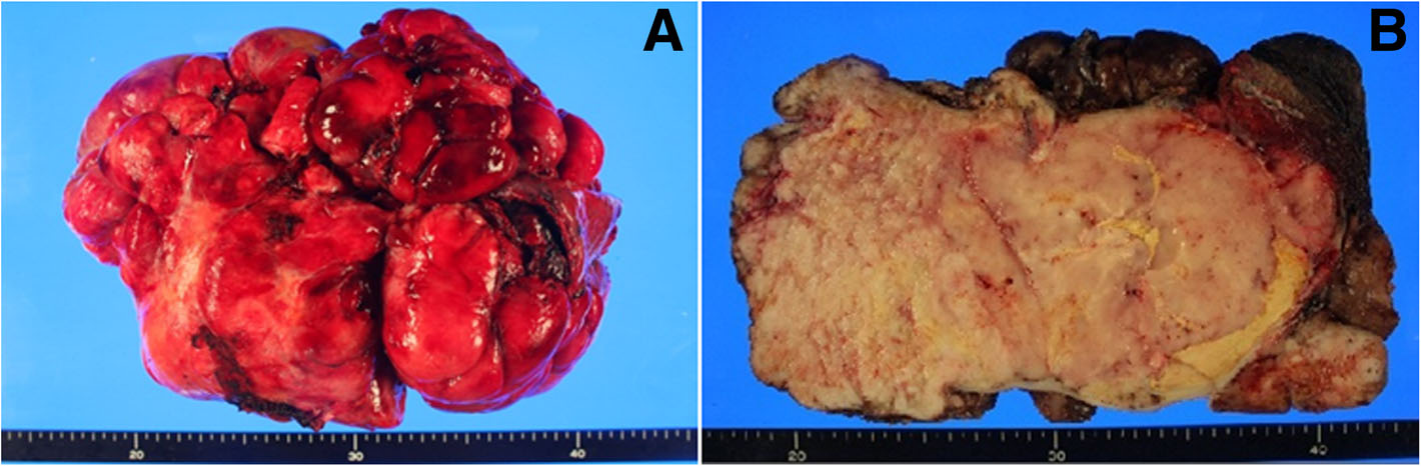
(**a**) Gross appearance of the tumor. (**b**) The cut section shows a well circumscribed encapulate white mass, mesuring 18 X 14 X 12 cm

**Fig. 4:**
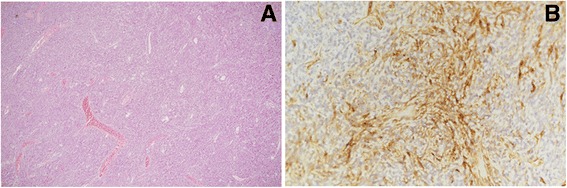
(**a**) Histology: proliferation of spindle cells with a highly vascular stroma 81 (×100). (**b**) Immunohistochemistry: diffuse membranous CD34 immunostaining ×200. Tumor cells were positive for CD34 and Bcl-2

The 18 × 15-cm mass weighing 2.05 kg was completely excised from normal adjacent tissues (Fig. 3 a-b). Histologically, pleomorphism of cells was not observed, but more than 5 mitoses were observed per 10 high magnification fields of view, and CD34 and bcl-2 positive findings were observed. In addition, cell necrosis and dense tissues were found, indicating a malignant tumor (Fig. 4 a-b).

The injection of glucose was no longer necessary postoperatively. On postoperative day 4, his blood glucose level was more than 300 mg/dL, so insulin was used to control his blood glucose level. The patient was discharged 10 days postoperatively without any complications. Five years later, he was healthy and no remarkable findings were observed upon examination of the chest CT scan.

## Discussion

SFTPs are very rare tumors; only about 800 cases have been reported since it was described by Lamperer and Rabin in 1931 until 2002 [4, 5]. The incidence rate of SFTPs is 2.8 of 10,000 persons [6]. Pleural solitary fibrous tumors mainly originate from submesothelial mesodermal tissues of the visceral pleura. SFTPs are mostly benign, and the ratio of malignant degeneration is approximately 12–15% [6]. SFTPs are a comparatively rare disease composed of about 5% of total pleural tumor developments [7]. Clinical manifestations of malignant cases are aggressive with local invasion, but metastasis is rare [8]. SFTPs most frequently occur in individuals aged in their 40s through 60s, and no sex difference in the incidence rate has been reported [6]. Usually, they are incidentally detected on a chest radiograph, as they do not have noticeable symptoms. About 50% of patients experience respiratory symptoms, such as pleuritic chest pain, coughing, and shortness of breath, or systemic symptoms, such as osteoarthritis, fever, and weight loss [7]. The paraneoplastic syndrome of hypoglycemia that occurs particularly due to insulin-like growth factor 2 (IGF-II), which is secreted from fibrous tumor cells, is called Doege-Potter syndrome. Only 45 cases of Doege-Potter syndrome have been reported from 1979 to 2011 [9]. Doege-Potter syndrome develops because of excessive secretion of IGF-II [6]. To make a histological diagnosis, an optical microscope and immunohistochemical staining are used. Histological sections show diverse shades of grey and a spiral, solid area mixed with soft phlegmatic components and spindle cells with patternless patterns, collagenous stroma with cellularity and hypocellularity tissue [10, 11] According to the standards suggested by England et al. [11], malignancy is determined in patients with invasive growth with an unclear boundary, high cell density, 4 or more mitoses per 10 high magnification fields of view, pleomorphism, and the presence of bleeding and necrosis. Cell necrosis and dense tissues are additional signs of a malignant tumor. In a more recent study, a huge size (>15 cm) and, elderly age (>55 years) were the predicted features for a poor prognosis [2].The definite treatment of SFTPs is surgical treatment with free resection margin [12]. Prognosis is mostly dependent on the possibility of excision, the size, number of division, and diversity of the cells, and the presence of necrosis [6]. Excluding the pedunculated tumor, which can be radically treated, the patients was diagnosed as having the disease about 10 year ago and did not undergo surgical resection, death occurred within 10 years [13]. Even if the tumor is malignant and the patient has polyposis with a clear boundary enabling excision, a good prognosis is possible [13]. Thus, prognosis is dependent on clear excision. The incidence rate of hypoglycemia associated with a fibrous tumor is very low: only 4% of 360 patients with a solitary fibrous tumor, according to a study conducted in 1981 [6]. Hypoglycemia has been confirmed to be associated with a large tumor size and high mitosis rate [8]. In addition, hypoglycemia disappears after the tumor is resected [12, 14]. For patients without symptoms, a plain radiograph is sufficient for follow-up. Reports on the effects of chemotherapy and irradiation therapy are very rare and limited [9, 10, 12]. As a result, operative management is the only solution for radical treatment.

## Conclusions

In general, if the disease fails to meet the criteria for malignancy, it is a benign disease. However, the histologically benign Doege-Potter syndrome can be an aggressive disorder that has a significant effect on daily life, with repeated occurrences of hypoglycemia. Therefore, we confirm that early operation and treatment of the Doege-Potter syndrome significantly affect the patients’ outcome.

## Abbreviations

CT: Computed tomography
D/W: Dextrose/water
IGF-II: Insulin-like growth factor 2
SFTPs: Solitary fibrous tumors of pleura
